# Differential miRNA Expression Profiling Reveals Correlation of miR125b-5p with Persistent Infection of Japanese Encephalitis Virus

**DOI:** 10.3390/ijms22084218

**Published:** 2021-04-19

**Authors:** Chih-Wei Huang, Kuen-Nan Tsai, Yi-Shiuan Chen, Ruey-Yi Chang

**Affiliations:** Department of Life Science, National Dong Hwa University, Hualien 97401, Taiwan; u791214@gmail.com (C.-W.H.); kuennant@usc.edu (K.-N.T.); r66142001@hotmail.com (Y.-S.C.)

**Keywords:** Japanese encephalitis virus, miR-125b-5p, persistent infection

## Abstract

MicroRNAs (miRNAs) play versatile roles in multiple biological processes. However, little is known about miRNA’s involvement in flavivirus persistent infection. Here, we used an miRNA array analysis of Japanese encephalitis virus (JEV)-infected cells to search for persistent infection-associated miRNAs in comparison to acute infection. Among all differentially expressed miRNAs, the miR-125b-5p is the most significantly increased one. The high level of miR-125b-5p in persistently JEV-infected cells was confirmed by Northern analysis and real-time quantitative polymerase chain reaction. As soon as the cells established a persistent infection, a significantly high expression of miR-125b-5p was readily observed. Transfecting excess quantities of a miR-125b-5p mimic into acutely infected cells reduced genome replication and virus titers. Host targets of miR125b-5p were analyzed by target prediction algorithms, and six candidates were confirmed by a dual-luciferase reporter assay. These genes were upregulated in the acutely infected cells and sharply declined in the persistently infected cells. The transfection of the miR125b-5p mimic reduced the expression levels of Stat3, Map2k7, and Triap1. Our studies indicated that miR-125b-5p targets both viral and host sequences, suggesting its role in coordinating viral replication and host antiviral responses. This is the first report to characterize the potential roles of miR-125b-5p in persistent JEV infections.

## 1. Introduction

Japanese encephalitis virus (JEV) is a mosquito-borne flavivirus belonging to the Flaviviridae family. The genome contains a single-stranded, positive-sense RNA of 10,976 nucleotides (nts) in length in the majority of JEV strains [1]. JEV exists in a zoonotic transmission cycle between vector mosquitoes and wading birds, with pigs serving as amplifying hosts. The *Culex tritaeniorhynchus* mosquito is the primary transmission vector. Human beings contract JEV when bitten by infected mosquitoes, which causes acute meningomyeloencephalitis and encephalitis. The average fatality rate is approximately 30%, and 20–30% of survivors exhibit permanent neuropsychiatric sequelae. JEV is responsible for more than 68,000 clinical cases, with approximately 13,600–20,400 deaths annually. Thus, JEV is a continuing public health threat in Eastern and Southern Asia [2]. It has been suggested that persistent infections in individual hosts might be important for virus survival [3].

Numerous studies have demonstrated that cellular and viral factors play important roles to establish persistent infection [3]. Persistent infection has been reported for many flaviviruses. Viral persistence can also be established in vitro in various cell lines [4]. We and others have shown that JEV could establish a persistent infection in both mosquito (C6/36) and mammalian (BHK-21) cells. In particular, JEV-derived defective interfering (DI) RNAs contribute to the establishment of persistent infections [5,6,7]. Though the detailed mechanism for persistent infections is not fully understood, it appears to be a balance between viral replication and the host’s defense system by either the modulation of the virus; gene expression or the modification of the host’s immune responses.

MicroRNAs (miRNAs) are small, single-stranded noncoding RNAs of approximately 21–23 nucleotides that regulate protein synthesis by targeting mRNAs for translational repression or degradation at the posttranscriptional level. miRNAs play major roles in many cellular processes [8] including apoptosis [9], oncogenesis [10], cell proliferation [11], differentiation [12], and inflammation [13]. Both host- and virus-derived miRNAs regulate viral and host gene expression [14,15]. The diverse biological roles of miRNAs, including the regulation of viral replication, host immune evasion, pathogenesis, and cellular transformation, are beginning to be uncovered [16,17,18].

The vast majority of DNA viruses and some RNA viruses produce their own viral miRNAs to counteract many host cellular processes and regulate the viral life cycle [19]. Several flavivirus-derived miRNAs have been reported [20]. For example, the deep sequencing of dengue virus 2 (DENV2)-infected mosquitoes revealed various viral small RNAs (vsRNAs). One of the vsRNAs, namely DENV-vsRNA-5 (derived from the viral 3′-UTR) specifically targets to viral NS1. The inhibition of DENV-vsRNA-5 miRNAs was found to lead to significant increases in viral replication [21]. The miRNA KUN-miR-1, derived from the 3′-UTR of the West Nile virus (WNV), targets the host transcription factor and facilitates WNV replication in mosquito cells [22], thus indicating that viral-derived miRNAs could induce the up- or down-regulation of viral replication.

Several host-derived miRNAs have been shown to modulate JEV infection in various ways. The upregulation of miR-19b-3p and miR-155 stimulated the production of inflammatory cytokines against viral infection [23,24]. Increasing the expression of miR-15b, miR-19b-3p, miR29b, and miR-155 has been found to be directly or indirectly involved in the regulation of the NF-κB signaling pathway, a key regulator of the immune response to infection [23,24,25,26]. MiR-124 attenuates JEV replication by targeting dynamin 2, a GTPase responsible for vesicle scission [27]. JEV infection was found to downregulate the expression of miR-33a-5p, which targets eukaryotic translation elongation factor 1A1 (EEF1A1), one of the components of the viral replication complex [28]. MiR-370 was also found to be downregulated in JEV-infected glioblastoma cells, which further mediated innate immunity-related genes [29]. These reports indicated that various miRNAs play versatile roles during the JEV life cycle. However, whether miRNAs are involved in persistent JEV infection remains unresolved.

To elucidate the molecular basis of miRNAs involved in persistent JEV infections, we compared the miRNA expression profiles between acutely and persistently infected BHK-21 cells. Several differentially expressed miRNAs were identified, of which miR-125b-5p was the most abundantly expressed in the persistently infected cells. We confirmed the miR-125b-5p expression in acutely and persistently infected cells by Northern analysis and RT-qPCR. The inverse correlation of the expression of targeted genes with the level of miR-125b-5p suggested that miR-125b-5p might be involved in the regulation of these genes in the persistently infected cells. These results indicated that the abundant expression of miR-125b-5p in persistently-infected cells correlated with the establishment and maintenance of a persistent infection.

## 2. Results

### 2.1. Comparison Analysis of miRNAs in Response to Acute and Persistent JEV Infection

To obtain the global miRNA expression profiles in comparison with acute and persistent infections of JEV, we performed the nCounter miRNA quantitative assay. RNA was isolated from the cell lysates of acute and persistent infections of JEV, as well as mock controls, by using the consistent purification technique described in the Section 4; simultaneous isolation was performed to avoid batch effects. The NanoString hybridization platform was used to identify the candidate miRNAs whose levels were significantly altered in response to acute and persistent JEV infections. The NanoString platform entails using microscopy to count fluorescently bar-coded probes, which allows for the detection of microRNAs without the amplification or introduction of position-dependent effects. We first screened the altered cellular miRNA levels in response to acute and persistent JEV infections, and then we compared them with the mock infection controls. The differential expression profiles of miRNAs exhibited more than two-fold difference for a specific miRNA were selected. Among the incorporated miRNAs in this assay, the levels of 45 miRNAs were significantly elevated and those of 197 miRNAs were reduced in the acutely infected cells in comparison to those of the mock controls. Similarly, the levels of 92 miRNAs significantly increased and 214 miRNAs decreased in the persistently infected cells compared to the levels detected in the mock controls. Subsequently, we queried the data set to identify individual miRNA species that were differentially expressed in response to acute and persistent infections. To fulfill this objective, we selected miRNAs tightly associated with JEV infections, in which the differential expression of miRNAs exhibited a more-than two-fold difference between the acute and persistent infections. A total of 31 miRNAs exhibited significantly differentiated expressions in the persistently infected cells. Among them, the levels of 14 miRNAs exhibited an at-least two-fold increase and those of 17 miRNAs were reduced compared to the levels of the acutely infected cells (Table 1). Notably, all these miRNAs were differentially expressed by at least two standard deviations above the background (by using the nCounter miRNA array system) in descending order and were believed to be closely associated with the persistent infection of JEV.

### 2.2. MicroRNA-125b-5p Is Highly Expressed in BHK-21 Cells Persistently Infected with JEV

**Figure 1:**
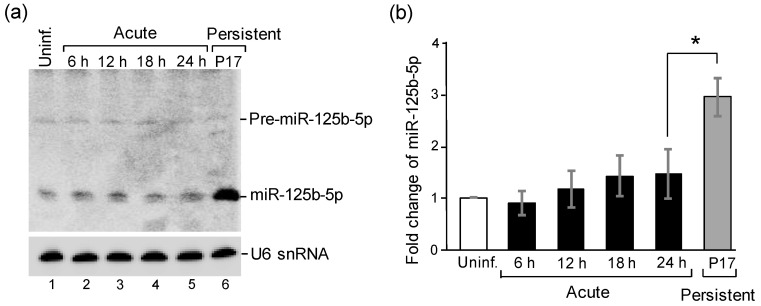
MiR-125b-5p is highly expressed in persistently JEV-infected cells. (**a**) Northern blot analysis of miR-125b-5p expression. Total RNAs were extracted from uninfected BHK-21 cells (lane 1), cells infected with JEV RP-9 at an MOI of 0.1 at the indicated times post-infection (hpi), and persistently infected cells at passage 17 (P17). Northern blot analysis was done using in vitro transcribed radiolabeled riboprobe specific to miR-125b-5p. The positions of precursor miR-125b-5p (pre-miR-125b-5p), mature miR-125b-5p (miR-125b-5p), and U6 snRNA are indicated. (**b**) The same RNAs were subjected to RT-qPCR analysis to determine the expression of miR-125b-5p. The relative fold change was normalized to endogenous U6 snRNA. Bars represent the mean ± SD (*n* = 3). *: *p*-value < 0.05 comparison between acute (24 hpi) and persistent infection (P17) was analyzed by Student’s *t*-test.

Among all differentially expressed miRNAs, miR-125b-5p was the most significantly increased one in the persistently infected cells compared to the acutely infected cells (Table 1). We further validated this result via Northern blot analysis. As shown in Figure 1a, miR-125b-5p exhibited a more upregulated pattern in the persistently infected cells compared to the uninfected and acutely infected cells. The RNAs were then subjected to RT-qPCR for the quantitative analysis of miR-125b-5p expression. As expected, there was a significant increase in the persistently infected cells (Figure 1b). The consistent results from the nCounter miRNA array, Northern blot analysis, and RT-qPCR indicated that miR-125b-5p was highly expressed in the persistently infected cells.

### 2.3. As Soon as the Cells Survived Infection, the High Level of miR-125b-5p Is Readily Detectable

**Figure 2:**
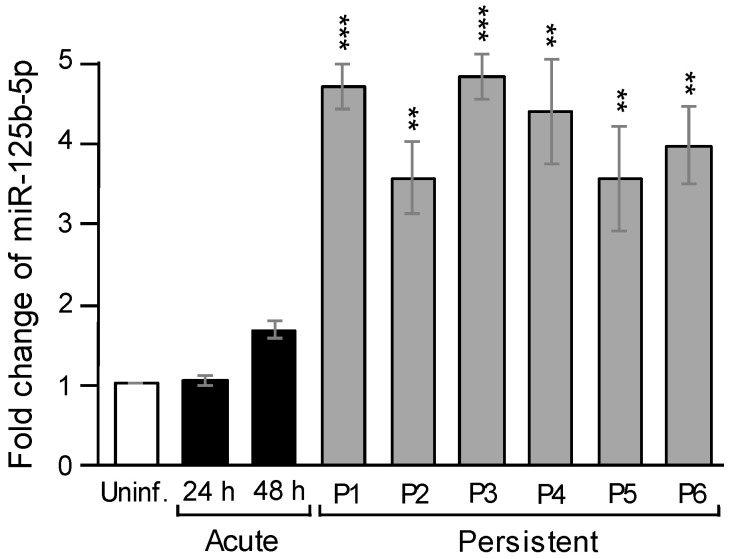
The miR-125b-5p was immediately elevated once established persistent infection. The total RNA was extracted from uninfected, acutely infected (24 and 48 hpi at an MOI of 0.1), and persistently infected cells from passages 1 to 6 (P1–P6). The RNA was subjected to an RT reaction and analyzed by RT-qPCR. The relative fold change was normalized to endogenous U6 snRNA. Bars represent the mean ± SD (*n* = 3). **: *p*-value < 0.01, ***: *p*-value < 0.001 comparison between acute (48 hpi) and persistent infection was analyzed by Student’s *t*-test.

Since the high level of miR-125b-5p was initially detected from the 17th passage of persistently infected cells, it was intriguing to examine the timing of miR-125b-5p upregulation after JEV infection. To do so, BHK-21 cells that survived JEV infection grew to confluence and were serially passaged (P1–P6). These cells were persistently infected, as demonstrated by the production of infectious viruses and an immunofluorescence assay with a virus-specific antibody (data not shown). RNAs were extracted from P1 to P6, as well as uninfected and acutely infected cells (namely 24 and 48 h post-infection). The miR-125b-5p expression level was measured by RT-qPCR. The expression of miR-125b-5p did not exhibit a difference between uninfected and acutely infected cells at 24 hpi, but it slightly increased at 48 hpi. The level of miR-125b-5p from each passage (P1–P6) of persistently infected cells was revealed to be three-to-five-fold higher than those in the acutely infected cells (Figure 2). These results indicated that as soon as the cells established a persistent infection, the miR-125b-5p expression level became significantly high, suggesting that a high level of miR-125b-5p expression may correlate with the establishment of a persistent infection.

### 2.4. Transfection of miR-125b-5p Mimic Inhibits Viral Replication

**Figure 3:**
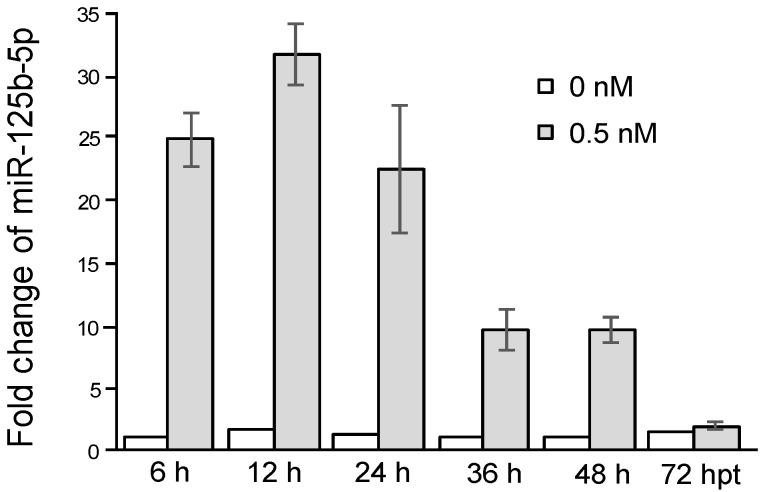
Transfected miR-125b-5p mimics lasted for 72 h. BHK-21 cells were mock-transfected or transfected with miR-125b-5p at 0.5 nM. Total RNA was extracted at 6, 12, 24, 36, 48, and 72 hpt. The RNA was subjected to an RT reaction and analyzed by RT-PCR. The relative fold change was normalized to endogenous U6 snRNA. Bars represent the mean ± SD (*n* = 3).

MiR-125b-5p has been reported to regulate both apoptosis and proliferation [30,31,32]. To evaluate the function of miR-125b-5p in JEV infection, we set up an experimental system by transfecting an miR-125b-5p mimic into BHK-21 cells. We first tested the cytotoxicity of the miR-125b-5p mimic at various concentrations and examined cell viability with an MTT assay using scrambled miRNAs as the negative control. The results showed that transfection with a dosage of less than 5 nM did not reveal cytotoxicity (data not shown). For safer and effective dosage, we chose 0.5 nM for the miR-125b-5p mimic for further studies. To measure the stability of the transfected miR-125b-5p in BHK-21 cells, the miR-125b-5p level was measured by RT-qPCR at 6–72 h post-transfection. The transfected miR-125b-5p mimic remained at a high level that was 23–30-fold higher than those mock transfection controls at 6 and 12 h post-transfection. Because the cells continued to grow and divide, the relative amounts of the transfected miR-125b-5p per cell, in addition to RNA stability, decreased with time. Nevertheless, it remained as high as eight-fold at 48 h post-transfection and slightly higher than that of the controls at 72 h post-transfection, suggesting that analyzing the effect of miR125b-5p by transfection could be measured within 72 h post-transfection (Figure 3).

**Figure 4:**
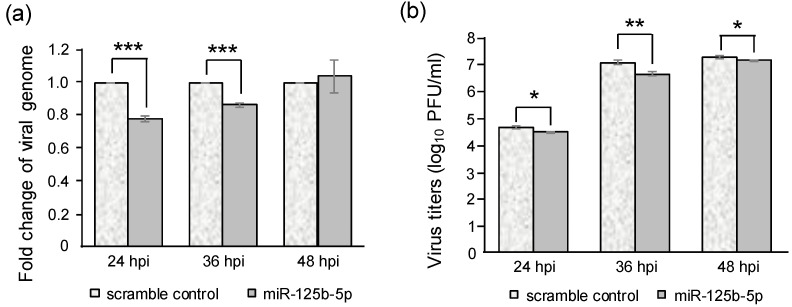
Transfecting excess amounts of miR-125b-5p inhibited JEV replication. BHK-21 cells were transfected with miR-125b-5p (0.5 nM) or a scramble control and then infected with JEV at an MOI of 0.1 at 24 h post-transfection. (**a**) Cytoplasmic RNA was extracted at the indicated time post-infection. The fold change of viral RNA was analyzed using RT-qPCR. (**b**) The supernatant was collected, and virus titers were determined by plaque assay. Bars represent the mean ± SD (*n* = 3). *: *p*-value < 0.05; **: *p*-value < 0.01; *** *p*-value < 0.001 compared with scramble control and analyzed by Student’s *t*-test.

To investigate whether miR125b-5p affects viral replication, BHK-21 cells were transfected with the miR-125b-5p mimic and then infected with JEV at 24 hpt. Cytoplasmic RNAs were isolated at 24, 36, and 48 hpi and subjected to RT-qPCR, and the supernatant was collected for plaque assays. The results showed that transfecting the miR-125b-5p mimics reduced the viral genome by 23% at 24 hpi and by 14% at 36 hpi compared to scramble controls (Figure 4a). As previously mentioned, because the transfected miR125b-5p mimics may not have been abundant at 72 hpt (Figure 3), the inhibition was not apparent at 48 hpi (72 hpt). Nevertheless, the supernatants containing virus fluids were reduced by 1.8-, 2.4-, and 0.7-fold in the miR-125b-5p-transfected groups in comparison with the controls as measured at 24, 36, and 48 hpi, respectively.

### 2.5. Targeting Genes of miR-125b-5p

**Figure 5:**
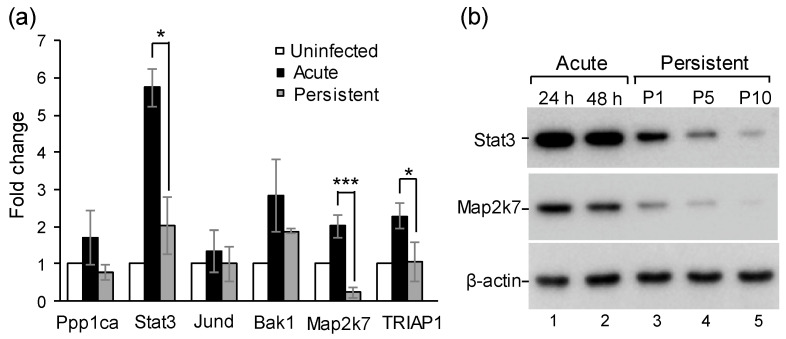
Downregulation of miR-12b-5p-targeted genes in the persistently JEV-infected cells. (**a**) Six predicted miR-125b-5p target genes were downregulated in persistently infected cells compared with the acutely infected cells. Total RNAs were extracted from uninfected, acutely infected, and persistently infected cells at 24 hpi (P10), and the amounts of the six genes were analyzed by RT-qPCR. The relative fold change was normalized to mock-infection. Bars represent the mean ± SD (*n* = 3). *: *p*-value < 0.05; ***: *p*-value < 0.001 comparison between acute and persistent infections was analyzed by a Student’s *t*-test. (**b**) Decreased expressions of both Stat3 and Map2k7 in persistently JEV-infected cells in comparison with acutely infected cells. Cell lysates from acute or persistent infection, as indicated on the top, were analyzed by 10% SDS-PAGE and Western blotting with antibodies, as indicated on the left.

To analyze the potential targets of miR-125b-5p, we used miRNA target prediction algorithms, including the TargetScan and miRTarBase software, for screening. Approximately 200 predicted genes were found to be the potential miR125b-5p targets. Among them, we focused on genes related to cell proliferation, immunity, and the cell cycle. The top six potential candidate genes, namely Ppp1ca, Stat3, Jund, Bak1, Map2k7, and Triap1, were selected (Table S1). The expression levels of these genes in the uninfected, acutely, and persistently infected cells were measured using RT-qPCR. Interestingly, the expression levels of the six predicted targeted genes were all upregulated in the acutely infected cells but downregulated in the persistently infected cells (Figure 5a). Among them, the expression levels of Stat 3, Map2k7, and Triap1 were significantly inhibited in the persistently infected cells. Stat3 is a signal transducer and activator of transcript 3, and it plays a key role in many cellular processes including cell growth and apoptosis [33,34]. Map2k7 is a member of the mitogen-activated protein kinase family, which is involved in signal transduction that mediates cellular responses to proinflammatory cytokines [35,36]. The expression levels of Stat3 and Map2k7 were further confirmed by Western blot analysis. As expected, the expression of both Stat3 and Map2k7 significantly decreased in the persistently infected cells (P1, P5, and P10) in comparison to the acutely infected cells (Figure 5b).

**Figure 6:**
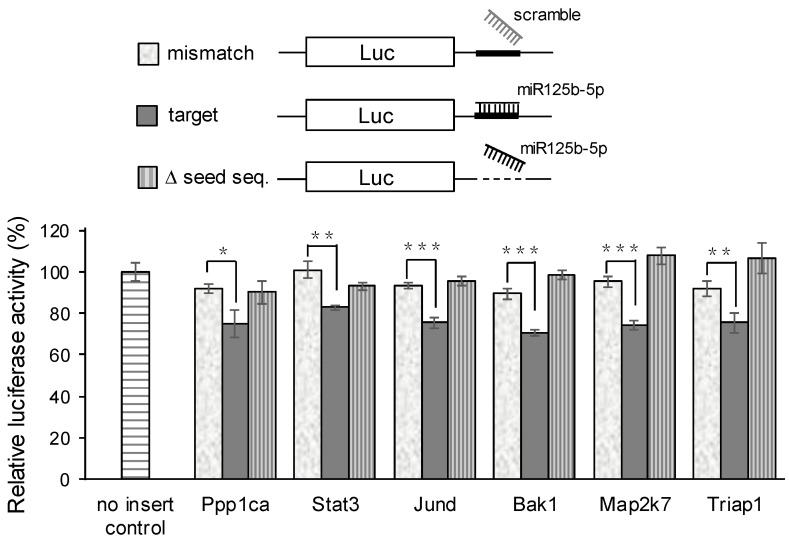
MiR-125b-5p targeted the six predicted genes, as demonstrated in luciferase reporter assays. Diagrams of the dual-luciferase reporter plasmids (pmirGLO vector; Table S3) and the microRNA (miRNA) are shown on the top. The activity was evaluated by the insertion of miRNA target sites at the 3′-UTR of the firefly luciferase gene (luc) and Renilla luciferase acting as a control reporter for normalization. The deletion of the miRNA target site was used as a negative control. BHK-21 cells were co-transfected with 100 ng of a luciferase reporter construct containing a gene target or the seed sequences deleted in conjunction with 0.5 nM of the miR-125b-5p mimic or the scramble miRNA as a negative control. After 24 h, cells were lysed for a dual-luciferase assay. Data are shown as means ± SD (*n* = 3). *: *p*-value < 0.05; **: *p*-value < 0.01; ***: *p*-value < 0.001 compared with the scramble control and analyzed by Student’s *t*-test.

To determine whether these genes are indeed targets of miR-125b-5p, we performed a dual-luciferase reporter assay by constructing plasmids containing the putative binding sites (from 3′-UTR of the six genes) for miR-125b-5p or its mutant derivatives by deleting the target seed sequences. Targeted DNAs or mutants were co-transfected with miR125b-5p mimics or scramble control miRNAs. As shown in Figure 6, the luciferase activity of all the six predicted genes markedly decreased when cells were co-transfected with the miR-125b-5p mimic but not in the scrambled control groups. Similarly, no inhibition was observed when the target binding regions were removed, suggesting that these genes are potential miR-125b-5p targets.

**Figure 7:**
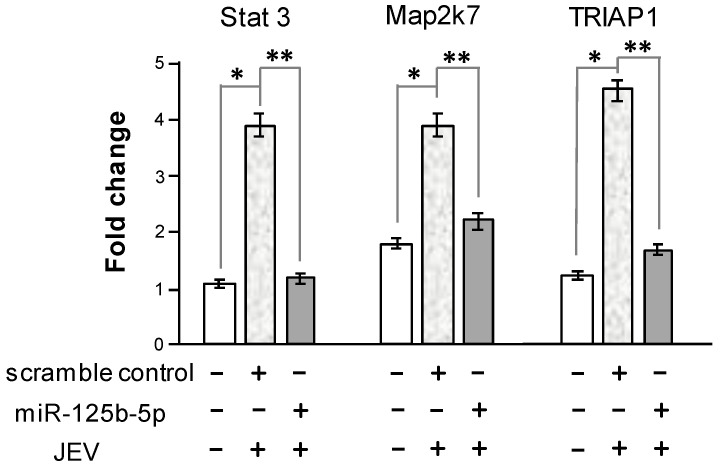
The overexpression of miR125b-5p downregulated the target genes. BHK-21 cells were transfected with miR-125b-5p or an miRNA scramble control. At 24 h post-transfection, cells were infected with JEV at an MOI of 0.1. Total RNA was extracted at 48 hpt and analyzed by RT-qPCR. The relative fold change was normalized to endogenous GAPDH RNA. *: *p*-value <0.05 in the comparison between mock infection and acute JEV infection analyzed by Student’s *t*-test. **: *p*-value < 0.01 in comparison between the miRNA scramble control and miR125b-5p transfection in the JEV-infected cells.

To further validate that miR-125b-5p affects the expression of target genes in JEV-infected cells, an miR-125b-5p mimic or scramble control was transfected into BHK-21 cells followed by JEV infection. Total RNAs were extracted, and the expression levels of Stat3, Map2k7, and TRIAP1 were measured using RT-qPCR. JEV infection caused the upregulation of these three genes. The transfecting of the miR-125b-5p mimic resulted in a significant reduction in Stat3, Map2k7, and TRIAP1 expression, while inhibition was not observed in those scramble control groups (Figure 7). These results suggested that Stat3, Map2k7, and TRIAP1 could be the direct targets of miR-125b-5p.

## 3. Discussion

Several miRNAs have been shown to play crucial roles during the JEV life cycle, but direct evidence for the role of miRNAs in persistence has yet to be defined. In this study, we observed that miRNAs were differentially expressed in persistently JEV-infected cells. Fourteen miRNAs were upregulated (Table 1), and among these, we focused on the most significantly increased one, miR-125b-5p. The upregulation of miR-125b-5p was not apparent after JEV infection in the acute phase and became highly abundant in the persistently infected cells (Figure 1 and Figure 2), indicating that miR-125b-5p correlates with viral persistence. Persistent infection has been previously reported for several flaviviruses [4,37,38]. It has been shown that DENV could establish a life-long persistent infection in mosquitoes [39]. A global miRNA profile in a mosquito C6/36 cell line persistently infected with DENV2 was examined, and the differential expression of miRNAs involved in several cellular pathways was found [40]. Nevertheless, miR125b-5p was not reported in these studies. Chen et al. examined the differentially expressed miRNA levels of JEV P3-infected human astrocytoma cells (U251) and found a slight reduction of miR-125b-5p in acute infection [28]. However, persistent infection was not examined in the study. Different cell types may contribute to distinct results. Furthermore, they obtained their miRNA profile with a high throughput miRNA sequencer, whereas we employed an nCounter miRNA array kit to obtain miRNA differentially expressed profiles. The NanoString nCounter platform relies on base pairing and does not require the amplification of target molecule [41]. We also noticed that the expression level of miR-125b-5p increased by more than 10-fold with the nCounter miRNA array and Northern blot analysis, but we only observed an approximately 3-fold increase with RT-qPCR (Table 1 and Figure 1). Some possible explanations for this discrepancy were the close sequence similarity between miR-125 family and that the hybridization may have also detected miR-125a-5p (5′-ucccugagacccuuuaaccuguga-3′; the base differences from miR-125b-5p are underlined).

The miR-125b family has been shown to regulate many cellular processes [42]. For example, miR-125b-5p was found to suppress proliferation and invasion in gastric cancer [43]. Conversely, miRNA-125b promotes glioblastoma proliferation and survival [44]. Additionally, miR-125b is a negative regulator of p53- and p53-induced apoptosis [45]. These controversial reports indicate that miR-125b may play dual roles in apoptosis that could either increase or reduce cell survival under different conditions or in various tissues by targeting versatile genes. It has been shown that miR125b-5p could prevent the apoptosis and necrosis of endothelial cells under oxidative stress by regulating the SMAD4-related pathway [46]. In contrast, miR-125b-5p was found to promote the apoptosis of synovial cells by targeting the synoviolin 1 (SYVN1) gene that encodes a protein involved in endoplasmic reticulum-associated degradation [47]. Because miR-125b-5p was highly expressed in the persistently infected cells, we hypothesized that miR-125b-5p may block apoptosis in persistently infected cells, thus contributing to the establishment of a persistent infection. We previously showed that persistently JEV-infected cells showed a slow growth rate [6]. Whether miR-125b-5p participates in the regulation of cell proliferation or the inhibition of cell apoptosis that may contribute to the slow growth rate of persistently infected cells remains to be determined.

Several regulators have been reported as the direct targets of miR-125b-5p [48,49,50,51,52]. Ppp1ca, Stat3, Jund, Bak1, Map2k7, and TRIAP1 were analyzed in this study. The expression levels of these genes were upregulated in acutely infected cells, whereas all decreased in the persistently infected cells when miR-125b-5p was upregulated (Figure 5a). These targets were further verified using a luciferase reporter assay (Figure 6). Furthermore, the transfecting of an miR-125b-5p mimic into the JEV-infected cells significantly reduced the expression Stat3, Map2k7, and TRIAP1 (Figure 7). These results indicated that these genes are indeed targets of miR-125p-5p.

Ppp1ca is one of the three catalytic subunits of protein phosphatase 1, which is essential for cellular metabolism, transcription, and cell cycle progression [53]. It has been shown that the upregulation of miR-125b induces tau hyperphosphorylation in Alzheimer’s disease by targeting Ppp1ca phosphatase [54]. Ppp1ca is also associated with protein kinase R (PKR), the host interferon (IFN)-induced antiviral and antiproliferative responsive protein [55]. Stat3 is a member of the STAT protein family, which is activated through tyrosine phosphorylation by cytokine receptor-associated kinases. Stat3 plays a key role in many cellular processes such as cell growth and apoptosis [56]. It has been shown that miR-125b-5p displays a tumor-suppressive role via targeting STAT3 [57]. These reports imply that both phosphorylating and dephosphorylating proteins could be regulated by miR-125b-5p and control the development of persistent infection.

The proper regulation of cell proliferation and apoptosis is likely required to maintain cell homeostasis. JunD is a versatile AP-1 transcription factor that exerts a pivotal role in cellular growth control [58]. Bak1 is a protein that acts as an apoptotic regulator and is involved in various cellular activities [59]. It has been shown that the mitochondrial antiviral signaling protein (MAVS) is critical for virus-induced apoptosis. MAVS recruits Map2k7 onto mitochondria and activates the apoptosis pathway [36]. Map2k7 was shown to be upregulated in triple-negative breast cancer (TNBC) and negatively correlated with the level of miR-125b [35]. The tumor protein p53-regulating inhibitor of apoptosis 1 (TRIAP1) is an apoptosis inhibitor that blocks the formation of the apoptosome and caspase-9 activation [60]. The antiapoptotic protein repressed by miR-125b-5p has indicated that apoptosis could be induced by TRIAP1 repression. Although the induction of apoptosis seems contradictory for persistent infection, miR-125b-5p is a multifunctional regulator and may simultaneously regulate numerous genes, suggesting that the establishment of persistent infection may involve several cellular pathways. TRIAP1 could be one of the negative regulators. Recently, TRIAP1 was demonstrated to be the target of miR-125b-5p in the regulation of apoptosis and inflammatory response in interleukin-1β-induced cells [30]. Nevertheless, further studies will be required to dissect the detailed mechanisms of miR-125b-5p in the regulation of these genes involved in persistent infection.

The miR-125b-5p level was found to be elevated in the serum of patients with chronic hepatitis B virus (HBV) infection, which resulted in the inhibition of the detection of HBV surface antigen [61]. Furthermore, the ectopic expression of miR-125b was found to inhibit the secretion of HBsAg and HBeAg by targeting the sodium channel, non-voltage-gated 1 alpha (SCNN1A) gene [62]. MiR-125b-5p also targets the LIN28B/let-7 axis to stimulate HBV replication at a post-transcriptional step [63]. It has also been shown that miR-125b reduced the replication of the porcine reproductive and respiratory syndrome virus (PRRSV) by negatively regulating the NF-κB pathway rather than by directly targeting the PRRSV genome [64]. These findings were somewhat similar to our observations. Though the miR-125b-5p was predicted to target the JEV genome in the regions of E, NS3, NS4B, and NS5 according to the ViTA database, the inhibition only led to a 14%–23% reduction on the viral genome and a 0.7–2.4-fold decrease of viral titers by transfecting the miR-125b-5p mimic (Figure 4). This inhibition may have resulted from the targeting host mRNAs reaching a balance between virus offense and host defense, thus potentially leading to the establishment of a persistent infection.

Numerous studies have suggested that both viral and host miRNAs in infected cells could indirectly regulate viruses through many pathways that could either enhance or reduce viral replication and determine the survival of infected cells [17]. We postulate that the possible mechanism(s) of viral persistence could be (i) the downregulation of viral RNA replication, (ii) the suppressing of the apoptosis of host cells, and (iii) the blocking host cell antiviral mechanisms. Since miR-125b-5p targets both viral genome and host gene expression, it could be a key regulator that provides a balance between virus replication and host antiviral responses. This balance has the added benefits of a persistent infection. Elucidating miRNA functions through the identification of targets could serve as a basis for future antiviral therapeutic development. We found that the high expression of the multifunctional miR-125b-5p correlated with persistent infection, which is crucial for elucidating JEV pathogenesis. In conclusion, our data suggested that the upregulation of miR125b-5p in persistently JEV-infected cells not only can regulate a number of genes involved in cellular signaling pathways associated with the apoptosis and controlling of cell growth but also inhibit viral replication. This is the first report characterizing miRNAs in persistent JEV infection.

## 4. Materials and Methods

### 4.1. Cells and Viruses

Baby hamster kidney (BHK-21) cells were cultured in RPMI 1640 supplemented with 2% fetal bovine serum (FBS; Gibco, Waltham, MA, USA) at 37 °C and 5% CO_2_. The JEV strain RP-9 (GenBank accession No. AF014161), a variant of NT109 originally isolated from *Culex tritaeniorhynchus,* was used in this study.

### 4.2. Plaque Assay

The titration of infectious virus was performed as previously described [6]. Briefly, serial 10-fold dilutions of the virus were added to a monolayer of BHK-21 cells in a 6-well plate. After 1 hr of adsorption, the unbound viruses were removed and the cells were gently washed with PBS and overlaid with a growth medium containing 1% SeaKem LE agarose (Lonza, Basel, Switzerland). After 48 hrs of incubation at 37 °C, the cells were fixed with 2% formaldehyde for 30 min, and the formaldehyde/agarose was gently removed. The fixed cells were stained with staining solution (0.5% crystal violet, 1.85% formaldehyde, 50% ethanol, and 0.85% NaCl) for 10 min and washed with deionized water. Viral titers were determined as plaque-forming units per milliliter (PFU/mL) for each viral stock.

### 4.3. Establishment of Persistently JEV-Infected BHK-21 Cell Line

The establishment of the persistently infected BHK-21 cells was carried out as previously described [6]. Briefly, the supernatant fluid containing DI particles from the 20th passage (P20) of persistently infected cells was used to infect fresh BHK-21 cells. Approximately 5% of the infected cells survived at 7 days post-infection. The re-fed surviving cells continued to grow to confluence by 20 days post-infection. Then, these cells were passaged and divided into two parts. One part of the cells was used for RNA extraction (P1), the other part of the cells continued for cell passage, and the RNAs were extracted at four-day intervals (P2–P10).

### 4.4. MicroRNA Profiling and Selection of Distinctive miRNAs Using a nCounter miRNA Array System

We employed the NanoString nCounter Mouse miRNA Expression Assay Kit (http://www.nanostring.com, accessed on 12 April 2021) to profile 600 mouse miRNAs. Equivalent amounts of RNA (100 ng) were prepared for the nCounter miRNA reactions according to the manufacturer’s instructions (NanoString Technologies, Seattle, WA, USA), and the detailed protocol was described previously [65]. Briefly, a specific DNA tag designed to normalize the T_m_s of each mature miRNA was ligated onto the 3’ end of the prepared small RNA samples and to provide unique identification for each miRNA. These RNAs were then hybridized with reporter probes, followed by their elongation and immobilization onto the imaging surface and analyzed by the nCounter digital analyzer. The normalization of expression counts of miRNAs was calculated by using the nSolver Analysis (version 1.1) software. Distinctively expressed miRNAs expressed from the nCounter analysis were selected. Briefly, each normalized miRNA was converted into a Log_2_ value. The Log_2_ value of the miRNA from the acute or persistent JEV infections with a more-than 2-fold or less than 0.5-fold difference than the Log2 value from the uninfected samples was grouped into JEV-regulated miRNAs. The miRNAs specifically expressed in the JEV-infected samples from persistent infections were selected if they had an approximate 2-fold expression level difference with the miRNA expression level of the sample from the acute infections.

### 4.5. RNA Extraction and Northern Blot Analysis of microRNAs

Small RNAs were extracted using specific isolation kits (mirVana, Thermo Fisher Scientific, Waltham, MA, USA) according to the manufacturer’s instructions. The amount of 15 μg of total RNA was dissolved in 15 μL of DEPC-treated water and mixed with 15 μL of RNA-loading dye (98% formamide, 10 mM EDTA, 0.1% xylene cyanol, and 0.1% bromophenol blue). The mixture was heated at 90 °C for 10 min and chilled rapidly on ice for 3 min. The denatured samples were separated by gel electrophoresis on a 12% polyacrylamide gel containing 8 M urea in TBE at 400 V for one and half hours. After electrophoresis, samples were transferred to a Hybond-N^+^ membrane (GE Life Sciences, Chicago, IL, USA) using a semi-dry transfer cell (Bio-Rad, Hercules, CA, USA). The membrane was crosslinked with a UV crosslinker. Riboprobes were synthesized by in vitro transcription. DNA templates used for in vitro transcription were made by annealing two synthetic oligonucleotides with complementary sequences of the T7 promoter (Table S2). Two oligonucleotides, the T7 promoter TOP primer (200 μM and 0.55 μL) and the miR-125b-5p primer (100 μM and 0.55 μL) were mixed, denatured at 95 °C for 3 min, and cooled down at room temperature for 20 min. RNA was transcribed using Riboprobe in vitro transcription systems (Promega, Madison, WI, USA), according to the manufacturer’s instructions. RNA transcripts were radiolabeled with [α-^32^P] UTP (10 μCi/μL, 5 μL) and incubated at 37 °C for 1 hr. RQ1 RNase-free DNase (Promega) was added to remove the DNA template and then incubated at 37 °C for 15 min. DEPC-treated H_2_O (80 μL) was added to a G-25 column (GE Life Sciences) and centrifuged at 700× *g* for 1 min to remove the preserved buffer. The reaction mixture was supplemented with DEPC-treated water to a final volume 100 μL, transferred to a G-25 column, and centrifuged at 700× *g* for 2 min to remove the unincorporated radioactivity. The riboprobe was transferred into the hybridization buffer or stored at −80 °C. The RNA sample on the membrane was prehybridized with a hybridization buffer (0.36 M Na_2_HPO_4_, 0.14 M NaH_2_PO_4_, 1 mM EDTA, 10% SDS, 25% formamide, and 0.1 mg/mL salmon testes DNA) for 1 h at 55 °C. The riboprobe was denatured at 95 °C for 5 min, chilled on ice for 3 min, and then transferred to hybridization buffer. The prehybridization buffer was removed and replaced with a fresh hybridization buffer containing a denatured riboprobe. Hybridization was carried out at 55 °C overnight. Post-hybridization wash was done by washing with buffer I (4× SSPE and 4% SDS) twice at 50 °C for 20 min and then twice with buffer II (0.1×X SSC and 0.5% SDS) at 50 °C for 5 min. The miR125b-5p-specific bands were exposed to a phosphorimaging plate (GE Life Sciences) for 1 day and visualized by a Typhoon FLA 9500 analyzer (GE Life Sciences).

### 4.6. qPCR

(I) For RT-qPCR of miRNAs, total RNA was reverse-transcribed by stem-loop pulsed reverse transcription [66]. Total RNAs (300 ng each) were mixed with 0.5 μL of 10 mM dNTPs and 1 μL of stem-loop RT primer (1 μM; Table S1), and then they were supplemented with DEPC-treated water to 12.65 μL. The mixture was heated to 65 °C for 5 min and chilled on ice for 2 min, followed by being briefly centrifuged. Four microliters of a 5× First-Strand cDNA buffer, 2 μL of 0.1 M DTT, 0.1 μL of RNase OUT (40 units/μL), and 0.25 μL of SuperScript III RT (200 units/μL, Invitrogen, Carlsbad, CA, USA) were added and incubated for 30 min at 16 °C, followed by pulsed RT for 60 cycles at 30 °C for 30 sec, 42 °C for 30 sec, and 50 °C for 1 sec. The mixture was then incubated at 85 °C for 5 min to inactivate the reverse transcriptase. (II) For the RT-qPCR of cellular mRNAs, the total RNAs and oligo (dT)_18_ (10 μΜ) were mixed to a final volume of 10 μL. The mixture was incubated at 65 °C for 5 min and chilled on ice. Four microliters of the 5× Reaction Buffer, 2 μL of dNTP (10 mM), and 0.5 μL of MMLV reverse transcriptase (200 units/μL; Promega) were added and incubated at 42 °C for 1 h. The reaction was terminated by heating at 70 °C for 10 min to inactivate the reverse transcriptase. Relative quantitative RT-PCR was performed using SsoFast™ EvaGreen Supermix (Bio-Rad, Hercules, CA, USA) according to the manufacturer’s instructions. One μL of synthesized cDNA, 0.5 μL of forward and reverse primers (10 μM each), 3 μL of ddH_2_O, and 5 μL of EvaGreen Supermix were gently mixed. The samples were heated at 98 °C for 2 min, followed by 40 cycles of 98 °C for 5 s, and 55 °C for 5 s using CFX96 Real-Time PCR Detection System (Bio-Rad, Hercules, CA, USA). The relative expression of miRNAs was normalized to that of internal control U6 small nuclear RNA within each sample using the 2^−∆∆Ct^ method. The quantification analysis of targeted genes was determined by the same method using PCR primers and 18S rRNA as an internal control, as described in Table S1. The accession number of the target genes and the control sequences used in this study are indicated in Table S1.

### 4.7. Transfection of miR-125b-5p and MTT Assay

BHK-21 cells (3 × 10^4^ cells/well) were seeded in 12-well culture plates and incubated overnight. The cells were transfected with the miR-125b-5p mimic (Exiqon, Germantown, MD, USA) at final concentrations of 0.2, 0.5, 1, 5, and 25 nM, with 1 μL of Lipofectamine 2000 reagent (Invitrogen) for each well in Opti-MEM, according to the manufacturer’s protocol. Cell viability was determined at 48 h post-transfection by the conversion of thiazolyl blue, 3-(4,5-dimethylthiazol-2-yl)-2,5-diphenyl tetrazolium bromide (MTT) to blue formazan crystals. An MTT reagent (500 μL; 0.5 mg/mL, Life Technologies, Carlsbad, CA, USA) was added into each well and incubated at 37 °C for 3 hrs. The reagent was removed, formazan crystals were dissolved in 500 μL dimethyl sulfoxide (DMSO, Sigma-Aldrich, St. Louis, MO, USA), and the optical density at 570 nm of 100 μL aliquots was measured by Multiskan™ GO Microplate Spectrophotometer (Thermo Fisher Scientific). Values were expressed as a percentage relative to those obtained in scrambled siRNA control. Assays were performed in triplicate.

### 4.8. Prediction and Cloning of miR-125b-5p Target Sequences

Bioinformatics software TargetScan (http://www.targetscan.org/, accessed on 12 April 2021) and miRTarBase (http://mirtarbase.mbc.nctu.edu.tw/index.php, accessed on 12 April 2021) were used to predict the potential targets of miR-125b-5p. Synthetic oligonucleotides containing the target sequences of predicted genes were listed in Table S1. Forward and reverse primers (1 μg/μL, 2 μL) of each gene were annealed in 46 μL of an Oligo Annealing Buffer (Promega) and heated at 90 °C for 3 min, followed by incubating at 37 °C for 15 min. The annealed oligonucleotide DNA fragments containing *Pme*I and *Xba*I restriction enzyme sites were cloned into the pmirGLO vector (Promega) by T4 DNA ligase (Yeastern Biotech, Taipei, Taiwan) (Table S3). All clones were validated by DNA sequencing.

### 4.9. Western Blot Analysis

Two significantly differentially expressed proteins were confirmed by Western blots. Equal amounts of proteins from acutely or persistently infected cells (10 μg) were separated on SDS-10% PAGE for Western blot analysis using anti-Stat3 (Cell Signaling Technology, Danvers, MA, USA), anti-Map2k7 (Sigma-Aldrich), or anti-β-actin (Sigma-Aldrich, St. Louis, MO, USA) antibodies. Signals were revealed using a chemiluminescence kit (ECL. Amersham Parmacia Biotech, UK) and visualized using a luminescent image analyzer (LAS-3000, Fujifilm).

### 4.10. Luciferase Reporter Assay

For luciferase reporter assay, BHK-21 cells (1 × 10^5^ cells/well) were cultured in 12-well plates and transfected with 100 ng/well of the indicated reporter constructs (pmirGLO vector alone, pmirGLO-containing target genes, or pmirGLO-containing mismatch of target genes) in conjunction with a 0.5 nM miR-125b-5p mimic (Exiqon) or 0.5 nM scrambled miRNA (Exiqon) as a negative control using 2 μL of Lipofectamine 2000 (Invitrogen) according to the manufacturer’s protocol. At 24 h post-transfection, the cells were washed with PBS and lysed with 250 μL of a Passive Lysis Buffer (Promega), and cell extracts were subjected to the luciferase assay using a dual-luciferase assay system (Promega). After brief centrifugation (10,000× *g* for 30 s), 20 μL of supernatant were transferred into a tube containing 100 μL Luciferase Assay Reagent II (LAR II) and mixed by pipetting. Firefly luciferase (FLuc) activities were determined in a 20/20 luminometer (Turner Biosystems). Subsequently, 100 μL of Stop & Glo Reagent were added to determine Renilla luciferase activity. Relative luciferase activity was calculated by normalizing the ratio of Firefly/Renilla luciferase.

### 4.11. Statistical Analysis

Changes in RT-qPCR, virus titers, and luciferase activity were analyzed by Student’s *t*-test. *p*-values of less than 0.05 were considered significant.

## Figures and Tables

**Table 1:** The list of up- and down-regulated expression of miRNAs detected from the mock, JEV acutely infected, and JEV persistently infected BHK21 cells.

miRNAs	Accession Number	Sequence (5′–3′)	A ^1^	P ^2^	P/A
**Upregulated ^3^**					
mmu-miR-125b-5p	MIMAT0000136	UCCCUGAGACCCUAACUUGUGA	0.03	3.91	3.88
mmu-miR-181a	MIMAT0000210	AACAUUCAACGCUGUCGGUGAGU	0.46	3.09	2.63
mmu-miR-30a	MIMAT0000128	UGUAAACAUCCUCGACUGGAAG	0.84	3.13	2.29
mmu-miR-34a	MIMAT0000542	UGGCAGUGUCUUAGCUGGUUGU	0.11	2.24	2.13
mmu-miR-100	MIMAT0000655	AACCCGUAGAUCCGAACUUGUG	−0.35	1.76	2.11
mmu-miR-140	MIMAT0000151	CAGUGGUUUUACCCUAUGGUAG	0.02	1.66	1.64
mmu-miR-2135	MIMAT00000744	UGAGGUAGUAGGUUGUGUGGUU	0.75	2.36	1.61
mmu-miR-804	MIMAT0004210	UGUGAGUUGUUCCUCACCUGGA	0.28	1.77	1.49
mmu-miR-425	MIMAT0004750	AAUGACACGAUCACUCCCGUUGA	−1.02	0.45	1.47
mmu-miR-708	MIMAT0004828	AAGGAGCUUACAAUCUAGCUGGG	0.76	2.15	1.39
mmu-miR-532-5p	MIMAT0002889	CAUGCCUUGAGUGUAGGACCGU	0.34	1.72	1.38
mmu-miR-194	MIMAT0000224	UGUAACAGCAACUCCAUGUGGA	−1.10	0.24	1.34
mmu-miR-210	MIMAT0017052	AGCCACUGCCCACCGCACACUG	−1.28	−0.09	1.19
mmu-miR-30e	MIMAT0000248	UGUAAACAUCCUUGACUGGAAG	1.16	2.26	1.10
**Downregulated ^4^**					
mmu-miR-367	MIMAT0017214	ACUGUUGCUAACAUGCAACUC	1.72	0.63	−1.09
mmu-miR-214	MIMAT0004664	UGCCUGUCUACACUUGCUGUGC	−1.23	−2.34	−1.11
mmu-miR-202-5p	MIMAT0004546	UUCCUAUGCAUAUACUUCUUU	2.51	1.33	−1.18
mmu-miR-376a	MIMAT0003387	GGUAGAUUCUCCUUCUAUGAGU	1.21	−0.01	−1.22
mmu-miR-2133	MIMAT0000738	UUAAUAUCGGACAACCAUUGU	5.06	3.64	−1.42
mmu-miR-34b-3p	MIMAT0004581	AAUCACUAACUCCACUGCCAUC	0.32	−1.26	−1.58
mmu-miR-338-3p	MIMAT0000582	UCCAGCAUCAGUGAUUUUGUUG	1.49	−0.13	−1.62
mmu-miR-361	MIMAT0000704	UUAUCAGAAUCUCCAGGGGUAC	0.01	−1.67	−1.68
mmu-miR-208b	MIMAT0017280	AAGCUUUUUGCUCGCGUUAUGU	0.59	−1.13	−1.72
mmu-miR-132	MIMAT0016984	AACCGUGGCUUUCGAUUGUUAC	−0.48	−2.22	−1.74
mmu-miR-15b	MIMAT0000124	UAGCAGCACAUCAUGGUUUACA	0.25	−1.52	−1.77
mmu-miR-2140	MIMAT0000753	ACAGUAGAGGGAGGAAUCGCAG	3.23	1.38	−1.85
mmu-miR-31	MIMAT0000538	AGGCAAGAUGCUGGCAUAGCUG	0.03	−2.07	−2.10
mmu-miR-490	MIMAT0017261	CCAUGGAUCUCCAGGUGGGU	0.58	−1.63	−2.21
mmu-miR-125a-3p	MIMAT0004528	ACAGGUGAGGUUCUUGGGAGCC	0.20	−2.45	−2.65
mmu-miR-1224	MIMAT0005460	GUGAGGACUGGGGAGGUGGAG	2.98	0.30	−2.68
mmu-miR-128	MIMAT0000140	UCACAGUGAACCGGUCUCUUU	0.60	−2.25	−2.85

^1^. Values represent the fold change (log_2_) of significantly up- or down-regulated miRNAs in cells acutely infected with JEV (A.) measured at 24 h post infection compared to mock infection. ^2^. The cells that survived acute infection were passaged every 5 days. The RNA was extracted at the 17th passage and subjected to RT-qPCR. Values represent the fold change (log_2_) of significantly up- or down-regulated miRNAs in cells persistently infected with JEV (P.) compared to the mock-infected cells. ^3^. Upregulation is defined as an expression level of miRNA of more than or equal to 2 folds detected in the persistent JEV infection in comparison with acute JEV infection. ^4^. Downregulation is defined as an expression level of miRNA of less than 2 folds detected in the persistent JEV infection in comparison to the acute JEV infection.

## Supplementary Materials

The following are available online at https://www.mdpi.com/article/10.3390/ijms22084218/s1, Table S1: Accession number of the genes and the sequences used in this study, Table S2: Oligonucleotides used in this study, Table S3: Plasmid DNA used in this study.
