# Supplementary material for: Differential miRNA Expression Profiling Reveals Correlation of miR125b-5p with Persistent Infection of Japanese Encephalitis Virus
Source: Int J Mol Sci. 2021 Apr 19;22(8):4218. doi: 10.3390/ijms22084218 (PMC8073291; doi:10.3390/ijms22084218)
Supplement: Supplementary file 1 [file ijms-22-04218-s001.zip › ijms-1186870-supplementary.pdf]

Table S1. Accession number of the genes or sequences used in this study.

**Name**	**Accession number**	**Gene function or purpose**
Ppp1ca	NM_031868	Cell-cycle regulators
Stat3	MMU06922	Regulation of cell growth and apoptosis
Jund	NM_010592	Modulate tumor angiogenesis, cellular differentiation, proliferation, and apoptosis
Bak1	NM_007523	Pro-apoptotic member of the Bcl-2 gene family
Map2k7	NM_001164172	Signal transduction mediating the cell responses to proinflammatory cytokines
TRIAP1	NM_026933	P53-inducible cell-survival factor
18S rRNA	NR_003278	As internal control in qPCR
U6 snRNA	NR_003027	As internal control in qPCR and northern blot

Table S2. Oligonucleotides used in this study.

**Oligonucleotide**[a]	**Forward Sequence (5'→3')**[b]	**Binding or target region**[c]
**Q-PCR primers**		
Ppp1ca-F	CAAGGATGTTCAAGGCTG	673-690
Ppp1ca-R	GTCCAAATCATGCTTGTG	729-752
Stat3-F	AAGACCAAGTTCATCTGTGT	2398-2417
Stat3-R	CAGCACCTTCACCGTTAT	2495-2512
Jund-F	AAAGAAACGGGAAGAAGAAA	2701-2720
Jund-R	GGATTACGGAACAGGAATG	2814-2832
Bak1-F	AGACCAACTGTAGGAGACT	1358-1376
Bak1-R	GGGTAGGAGATAGGAGGG	1440-1457
Map2k7-F	CGCCTCTTCATCCTATTCTC	2964-2983
Map2k7-R	CTACTTCATTCCGCCTCC	3071-3088
TRIAP1-F	GGTGTTCTCCTCCATTCT	351-368
TRIAP1-R	TGATGCCAGACCATAGTT	467-484
18S-rRNA-F	CGCGGTTCTATTTTGTTGGT	878-897
18S- rRNA-R	AGTCGGCATCGTTTATGGTC	1077-1096
**miRNA Q-PCR primers**		
miR-125b-5p stem-loop RT primer	GTCGTATCCAGTGCAGGGTCCGAGGTATTCGCA CTGGATACGACTCACAA	
miR-125b-5p-F	TCGGCTCCCTGAGACCCTAA	
miR-125b-5p-R	GTGCAGGGTCCGAGGT	
U6-F	CTCGCTTCGGCAGCACA	
U6-R/RT primer	AACGCTTCACGAATTTGCGT	
**Primers used for cloning of target genes**		
Ppp1ca-target-F	*aaac***gaattc**GGCCAAGGCTGCAGCTCAGGGC*t*	1241-1262
Ppp1ca-target-R	*ctaga*GCCCTGAGCTGCAGCCTTGGCC**gaattc***gttt*	
Ppp1ca-Δ-F	*aaac***gaattc**GGCCAAGGCTGCAG*t*	
Ppp1ca-Δ-R	*ctaga*CTGCAGCCTTGGCC**gaattc***gttt*	
Stat3-target-F	*aaac***gaattc**TCTGGGGCTGGTGTTGTACCTCAGGGG*t*	4179-4205
Stat3-target-R	*ctaga*CCCCTGAGGTACAACACCAGCCCCAGA**gaattc** *gttt*	
Stat3-Δ-F	*aaac***gaattc**TCTGGGGCTGGTGTTGTAC*t*	
Stat3-Δ-R	*ctaga*GTACAACACCAGCCCCAGA**gaattc***gttt*	
Jund-target-F	*aaac***gaattc**CTCCGAGTAGGGGCTCTAAGGGG*t*	2533-2555
Jund-target-R	*ctaga*CCCCTTAGAGCCCCTACTCGGAG**gaattc***gttt*	

Jund-Δ-F	*aaac***gaattc**CTCCGAGTAGG*t*	
Jund-Δ-R	*ctaga*CCTACTCGGAG**gaattc***gttt*	
Bak1-target-F	*accc***gaattc**TTGGCGGCTGGACTCTCAGGGA*t*	1477-1498
Bak1-target-R	*ctaga*TCCCTGAGAGTCCAGCCGCCAA**gaattc***gttt*	
Bak1-Δ-F	*aaac***gaattc**TTGGCGGCTGGACTCGCGGA*t*	
Bak1-Δ-R	*ctaga*TCCCGGAGTCCAGCCGCCAA**gaattc***gttt*	
Map2k7-target-F	*aaac***gaattc**TGTCTCTCTTTGATCTCAGGGG*t*	2104-2125
Map2k7-target-R	*ctaga*CCCCTGAGATCAAAGAGAGACA**gaattc***gttt*	
Map2k7-Δ-F	*aaac***gaattc**TGTCTCTCTTTGA*t*	
Map2k7-Δ-R	*ctaga*TCAAAGAGAGACA**gaattc***gttt*	
TRIAP1-target-F	*aaac***gaattc**GACCTCTTTTTCCTCTCAGGGA*t*	601-622
TRIAP1-target-R	*ctaga*TCCCTGAGAGGAAAAAGAGGTC**gaattc***gttt*	
TRIAP1-Δ-F	*aaac***gaattc**GACCTCTTTTTCCTA*t*	
TRIAP1-Δ-R	*ctaga*TAGGAAAAAGAGGTC**gaattc***gttt*	
**Primers used for riboprobe preparation**		
T7 TOP-F primer	GATAATACGACTCACTATAGGGAGA	
U6-R primer	AAAAAAAAGGATGACACGCAAATTCGTGTCTCC CTATAGTGAGTCGTATTATC	69-90
miR-125b-5p-R primer	AAAAAATCCCTGAGACCCTAACTTGTGATCTCCC TATAGTGAGTCGTATTATC	1-22
**Synthesized microRNAs**		
mmu-miR-125b-5p mimic	UCCCUGAGACCCUAACUUGUGA	
miRNA scramble control	ACGTCTATACGCCCA	

a. Polarity of oligonucleotide with plus or minus sense is indicated by forward (F), or reverse primer (R), respectively. Deletion of seed sequences is denoted by “Δ”.

b. Non-binding region sequences are shown in lowercase. *Pme*I and *Xba*I restriction enzyme sites used for cloning are in italics. *Eco*RI site used for clone confirmation is in boldface. The underlined sequences represent seed sequences of target region used for deletion constructs. The T7 promoter is shown in shaded gray.

c. Numbers correspond to nucleotide positions in accession number as described in Table S2.

Table S3. Plasmids used in this study.

Plasmid DNA	Length (bp)	Description
pmirGLO Vector	7350	The pmirGLO Dual-Luciferase miRNA Target Expression Vector (promega) is designed to quantitatively evaluate miRNA activity by inserting miRNA target sites at 3´ of the firefly luciferase gene. *Renilla* luciferase acting as a control reporter for normalization and selection.
pmirGLO-Ppp1ca target	7356	3'-UTR of Ppp1ca gene at nt 1241-1262 was cloned into pmirGLO vector.
pmirGLO-Ppp1ca Δ	7348	Seed region at nt 1255-1262 was deleted form pmirGLO-Ppp1ca target
pmirGLO-Stat3 target	7361	3'-UTR of Stat3 gene at nt 4179-4205 was cloned into pmirGLO vector.
pmirGLO-Stat3 Δ	7353	Seed region at nt 4198-4205 was deleted from pmirGLO-Stat3 target.
pmirGLO-Jund target	7357	3'-UTR of Jund gene at nt 2533-2555 was cloned into pmirGLO vector.
pmirGLO-Jund Δ	7345	Seed region at nt 2544-2555 was deleted from pmirGLO-Jund target.
pmirGLO-Bak1 target	7356	3'-UTR of Bak1 gene at nt 1477-1498 was cloned into pmirGLO vector.
pmirGLO-Bak1 Δ	7354	Seed region at nt 1492-1497 of Bak1 was deleted from pmirGLO-Bak1 target
pmirGLO-Map2k7 target	7356	3'-UTR of Map2k7 gene at nt 2104-2125 was cloned into pmirGLO vector.
pmirGLO-Map2k7 Δ	7347	Seed region at nt 2117-2125 was deleted from pmirGLO-Map2k7 target
pmirGLO-TRIAP1 target	7356	3'-UTR of TRIAP1 gene at nt 601-622 was cloned into pmirGLO vector.
pmirGLO-TRIAP1 Δ	7349	Seed region at nt 615-621 was deleted from pmirGLO-TRIAP1 target
